# Artificial Intelligence Across the Prostate Cancer Pathway: Screening, Imaging, Pathology, and Biomarkers

**DOI:** 10.7759/cureus.96226

**Published:** 2025-11-06

**Authors:** Muhammad Rakib Hasan, Nazeer Ibraheem, Mohammad Ekhlasur Rahman, Rezuana Tamanna

**Affiliations:** 1 Urology, Watford General Hospital, West Hertfordshire Hospitals NHS Foundation Trust, Watford, GBR; 2 Urology, The Royal Wolverhampton NHS Trust New Cross Hospital, Wolverhampton, GBR; 3 General Surgery, Craigavon Area Hospital, Northern Ireland Medical &amp; Dental Training Agency (NIMDTA), Portadown, IRL

**Keywords:** ai, artificial intelligence, prostate cancer, prostate imaging, screening

## Abstract

Artificial intelligence (AI) has made great changes to prostate cancer screening and early detection across biomarkers, imaging, and pathology. On micro-ultrasound (micro-US), AI improves discrimination and raises specificity at comparable sensitivity versus clinical models, while multimodal magnetic resonance imaging-transrectal US (MRI-TRUS) AI achieves higher specificity at matched sensitivity. Liquid-biopsy programs combine fragmentomics with ctDNA and cell-free mRNA interpreted by AI, enabling noninvasive risk stratification and clinical feasibility. In imaging, AI for MRI matches or exceeds expert radiologists in large reader studies and MRI benchmarks; commercial tools show robust patient- and lesion-level performance. Quantitative pipelines (e.g., automated tissue-composition metrics) aid equivocal Prostate Imaging-Reporting and Data System (PI-RADS 3) lesions with PSA density, and AI-derived intraprostatic tumor volume offers independent prognostic value. Multimodal fusion of MRI with TRUS boosts detection, and automated prostate-specific membrane antigen (PSMA) PET/CT algorithms quantify tumor burden and support longitudinal response tracking. In pathology, clinical-grade AI automates cancer detection and Gleason grading, cutting reading time, ancillary tests, and second-opinion requests, while supporting integrative prognostic models. Downstream, AI accelerates radiotherapy planning, guides focal therapies and surgical margins, personalizes systemic therapy, and enables early post-treatment monitoring. Translation still requires rigorous, prospective, multi-site validation.

## Introduction and background

Prostate cancer is a common disease that significantly contributes to morbidity and mortality in male patients [1]. It has the highest incidence in Northern Europe, Australia/New Zealand, the Caribbean, and North America, and is the most common male cancer in about two-thirds of countries worldwide [2]. In the U.S., three cancers, prostate, lung/bronchus, and colorectal, make up about 48% of new male cases. Prostate cancer alone accounts for 29% of diagnoses in 2024 and is the second leading cause of cancer death in men [3].

Prostate cancer diagnosis relies on a core needle biopsy (CNB) of the prostate, typically performed on patients with elevated prostate-specific antigen (PSA) levels in their blood or an abnormal digital rectal examination. The subsequent morphological examination of prostatic tissue by a pathologist can be a time-consuming and demanding task, requiring sustained concentration to identify subtle changes in glandular architecture and cellular atypia, the complexity of which varies with biopsy modality and the number of cores obtained [4]. Artificial intelligence (AI) is used in pathology because it can extract quantitative, high-dimensional features from digital slides using predefined computational methods [5].

AI is transforming prostate cancer care by boosting diagnosis, treatment planning, and management. Advanced imaging and digital pathology models accurately detect lesions and help predict outcomes, leading to more precise, data-driven decisions [6,7]. 

Multiple AI tools have been developed for histopathology assessment, diagnostic imaging interpretation, and risk stratification, with some achieving FDA approval for clinical use [5,8]. Research has particularly focused on radiomics, pathomics, and treatment outcome prediction, showing improved accuracy and efficiency in prostate cancer detection and management [9]. Despite promising results across imaging modalities and histopathology, implementation challenges remain, including validation requirements and integration into clinical workflows [10].

## Review

Methods

This narrative review examined AI across the prostate cancer pathway, screening, imaging, pathology, biomarkers, and treatment planning. We searched Medical Literature Analysis and Retrieval System Online (MEDLINE)/PubMed, Excerpta Medica database (Embase), Web of Science, IEEE Xplore, arXiv, and ClinicalTrials.gov (January 2019-October 2, 2025). Queries combined prostate cancer with AI, machine learning, radiomics, pathomics, PSA, MRI, transrectal ultrasound (TRUS), prostate-specific membrane antigen (PSMA) PET/CT, biopsy, radiotherapy planning, and focal therapies. 

Eligible studies evaluated AI, or "machine learning applied to prostate cancer screening, risk stratification, diagnostic imaging, histopathology, biomarker discovery, treatment selection, or planning, reporting clinical or technical outcomes. We included prospective, retrospective, multicenter evaluations, systematic reviews, and guidelines. We excluded single-case reports, editorials, non-prostate work, and purely technical papers lacking clinical validation. Preprints and abstracts were included when methods were transparent and relevant. 

We extracted data on study design, population, imaging/assay modality, ground truth, and dataset size/splits; documented external validation, calibration, and decision thresholds; and recorded performance metrics including AUC [11], sensitivity/specificity [12], positive predictive value (PPV)/negative predictive value (NPV) [13], and the Dice coefficient [14]. Where available, we conducted decision-curve analysis [15] and summarized time-to-event outcomes with Kaplan-Meier and Cox proportional-hazards methods [16-20]. Risk of bias was assessed using Quality Assessment of Diagnostic Accuracy Studies 2 (QUADAS-2) for diagnostic accuracy studies and Prediction model Risk Of Bias ASsessment Tool (PROBAST) for prognostic models [21,22], with adherence checks for Transparent Reporting of a multivariable prediction model for Individual Prognosis Or Diagnosis (TRIPOD), Consolidated Standards of Reporting Trials for Artificial Intelligence (CONSORT-AI), and Developmental and Exploratory Clinical Investigation of DEcision support systems driven by Artificial Intelligence (DECIDE-AI) [23-25]. Given heterogeneity in populations, modalities, and reporting, we used narrative synthesis following Synthesis Without Meta-analysis (SWiM) guidance [26], emphasizing model validity and calibration [18], clinically meaningful thresholds [19], and robust internal-external/external validation [20].

AI in prostate cancer screening and early detection

The future of prostate cancer screening is significantly improved and changed as AI improves. Traditional PSA-driven pathways are hampered by a low positive biopsy yield of 25% among men biopsied for elevated PSA, contributing to overbiopsy and overdiagnosis; AI-enabled approaches can better prioritize imaging and biopsy, lowering patient burden [27]. The specificity of PSA testing can vary considerably, with some studies reporting ranges between 6% and 66%, further complicating its diagnostic utility [28].

AI applied to micro-ultrasound (micro-US) shows strong promise for sharpening diagnostic work-ups in men evaluated for suspected prostate cancer. In a retrospective, single-center cohort of 145 men referred for biopsy, investigators trained a self-supervised convolutional autoencoder to extract features from 2D micro-US slices and used a random-forest classifier; patient-level calls required ≥8 consecutive positive slices. Against a clinical model using PSA, digital rectal examination, prostate volume, and age, the AI-interpreted micro-US model achieved a higher area under the receiver operating characteristic curve (AUROC) (0.871 vs. 0.753). With a fixed probability threshold (0.15), the AI model maintained high sensitivity (92.5%) while markedly improving specificity (68.1%) compared with the clinical model’s 96.2% sensitivity but only 27.3% specificity; precision (77.8% vs. 61.4%), F1 (84.5% vs. 74.9%), and accuracy (81.4% vs. 64.8%) were all superior. These gains suggest the potential to curb unnecessary biopsies without compromising detection of clinically significant disease (Gleason ≥3+4), while also reducing operator-dependence in micro-US interpretation. Nonetheless, the retrospective design, single-center setting, and lack of external validation warrant cautious generalization and motivate prospective, multi-center studies [29].

When compared directly with routine clinical MRI readings, the multimodal AI achieved higher specificity (88% vs. 78%) with equivalent sensitivity (79%) and a higher AUC (0.90 vs. 0.79); concordant findings were also highlighted in an AUA 2025 abstract reporting that a multimodal MRI-TRUS AI model exceeded radiologist performance [30].

Liquid biopsy using circulating tumor DNA (ctDNA) is rapidly advancing as a non-invasive screening approach, with AI increasingly used to interpret complex fragmentomic and genomic signals for risk stratification [31-33].

The FateAI platform applies AI models to circulating DNA fragmentomics and has shown early, multi-cancer detection promise in preclinical/early clinical datasets [34]. Methodological innovations include precision liposomal priming agents that transiently reduce macrophage-mediated clearance to enhance ctDNA recovery, thereby boosting the sensitivity of early-stage detection [35]. Genomic biomarkers integrated with machine learning show promise for addressing the heterogeneity of prostate cancer across populations by improving risk assessment beyond PSA alone [36].

**Figure 1:**
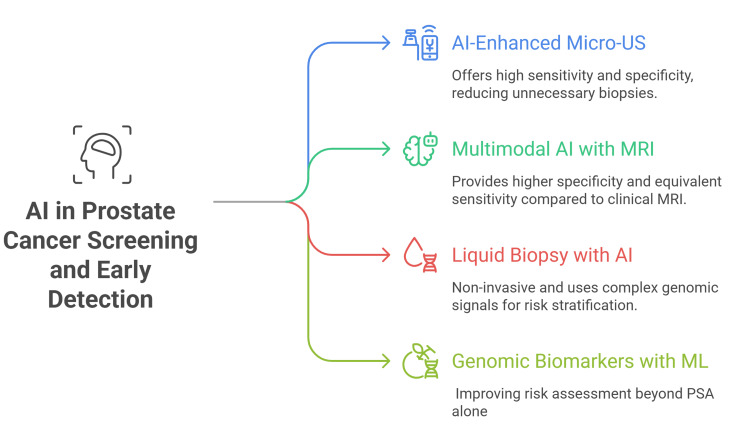
AI in prostate cancer screening and early detection This author-designed figure is based on references [27–38]. Micro-US: micro-ultrasound; PSA: prostate-specific antigen; ML: machine learning

A multicenter effort reported a 25-gene, blood-based mRNA panel (GeneVerify) with strong discriminative performance for prostate cancer detection (AUC 0.906; sensitivity 90%; specificity 91%) and proposed clinical use across screening, early diagnosis, prognosis, and monitoring [37]. An oncology-meeting abstract described real-time clinical validation of the same cell-free mRNA assay for screening and early detection, supporting feasibility in practice (Figure 1) [38].

AI in diagnostic imaging for prostate cancer

Multiparametric MRI enhanced by AI represents a paradigm shift in prostate cancer detection, addressing the inherent limitations of traditional Prostate Imaging-Reporting and Data System (PI-RADS) interpretation. Recent large-scale studies demonstrate AI systems achieving superior performance compared to expert radiologists, with AUC values of 0.91 versus 0.86 for human readers. These advances promise to transform clinical workflows and diagnostic accuracy [39,40].

In the Twilt JJ et al. International Multireader Diagnostic Study [39] (61 readers; 360 men), investigators compared prostate MRI readings with and without a validated Prostate Imaging-Cancer AI (PI-CAI AI) assistant. Clinically significant cancer prevalence was 34%. AI raised AUROC from 0.882 to 0.916 (+3.3%), increased sensitivity from 94.3% to 96.8% (+2.5%), and improved specificity from 46.7% to 50.1% (+3.4%) at PI-RADS ≥3. Stand-alone AI achieved AUROC 0.947. Gains were larger for nonexperts than for experts across operating points. PI-RADS distributions were similar, but AI reduced clinically significant prostate cancer (csPCa) prevalence in PI-RADS 1-2. Authors conclude AI assistance meaningfully improves csPCa diagnosis.

Rajeev et al.'s [40] study compared a deep-learning system with radiologists for detecting csPCa on biparametric MRI. Using 10,207 scans from 9,129 patients, with 2,440 GG≥2 cancers and ≥3-year follow-up, AI was benchmarked against 62 blinded experts and standard-of-care readings. AI achieved an AUC of 0.91 versus experts’ 0.86 (P<.001), yielded 50.4% fewer false positives and 20% fewer GG1 detections, and had 0.1% lower specificity than clinical readings; neither missed significant cancers. Authors support AI as a complementary tool pending prospective validation [40].

**Figure 2:**
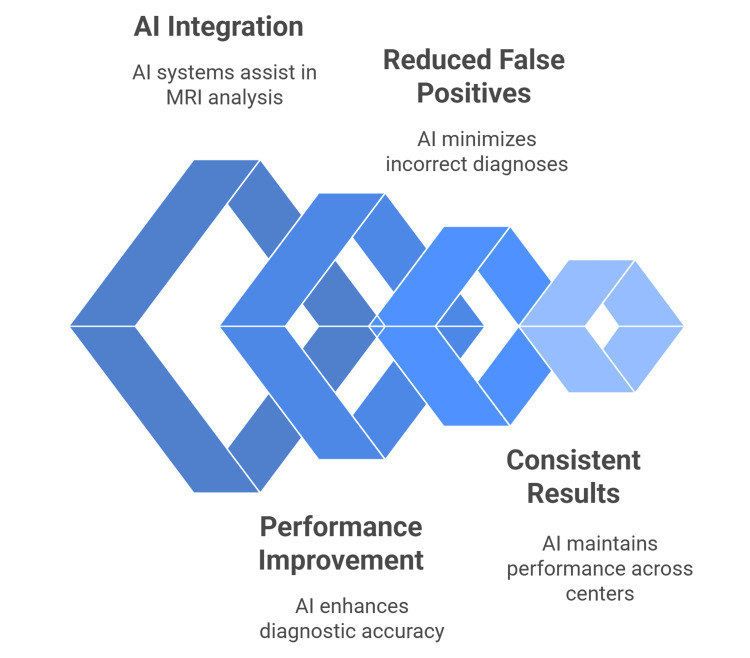
AI in diagnostic imaging for prostate cancer This author-designed figure is based on references [39–50].

A commercially available AI algorithm showed robust performance in a three-center benchmarking study. At the patient level (PI-RADS ≥3), AI achieved 91% sensitivity and 57% specificity for detecting csPCa. At the lesion level (PI-RADS ≥4), AI reached 78% specificity (vs. radiologists’ 70%), albeit with lower sensitivity (81% vs. 90%). Performance was consistent across the three university hospitals and standardized 3Tesla (3T) MRI protocols (Figure 2) [41].

Advanced Quantitative and Multimodal Imaging 

Hybrid multidimensional MRI tools incorporating AI demonstrate superior cancer detection compared to conventional PI-RADS assessment by expert radiologists. Prospective validation of automated tissue composition analysis using three-compartment modeling achieved higher diagnostic performance than traditional multiparametric MRI (mpMRI) interpretation. These systems identify regions with elevated epithelium (>40%) and reduced lumen (<20%), providing objective quantitative assessment beyond subjective visual interpretation [42].

PI-RADS category 3 lesions represent a critical diagnostic challenge where AI demonstrates particular value in reducing unnecessary biopsies. Combining AI analysis with PSA density achieves a sensitivity of 77.8% and a NPV of 93.1% for clinically significant cancer. This multimodal approach could reduce biopsies in 83.3% of patients with equivocal lesions while maintaining high cancer detection rates [43].

AI-derived intraprostatic tumor volume on mpMRI provided independent prognostic information beyond standard clinical and radiologic factors. The segmentation model was trained to delineate PI-RADS 3-5 lesions, and its volumetric (V_AI) predicted metastasis after treatment. For radical prostatectomy patients, five-year metastasis AUC was 0.89 for V_AI vs. 0.79 for risk defined by the National Comprehensive Cancer Network (NCCN; numerically higher; comparison not statistically significant). In the combined radiation therapy cohort, seven-year metastasis AUC was 0.84 for V_AI vs. 0.74 for NCCN (P = .02), indicating better discrimination [44].

Deep learning on multiparametric MRI, utilizing a ResNet50 feature extractor with multi-head attention, has demonstrated strong performance, achieving an AUC of 0.89 (PR-AUC of 0.91) by fusing T2-weighted, diffusion-weighted, and dynamic contrast-enhanced sequences for comprehensive tissue characterization [45]. Complementing this, a multimodal fusion framework that integrates MRI and TRUS further improves detection compared with unimodal models and even radiologists on key endpoints, underscoring the value of cross-modality AI [4].

Automated AI methods for PSMA PET-CT can quantify tumor burden and monitor response. In one study, a fully automated algorithm achieved sensitivities of 85% for prostate tumor/recurrence, 91% for lymph node metastases, and 61% for bone metastases, with strong agreement with manual quantification (r = 0.62-0.96 for total lesion volume (TLV)/total lesion uptake (TLU)). The aPROMISE (automated PROstate Cancer Molecular Imaging Standardised Evaluation) platform further supports fully automated longitudinal lesion tracking for treatment-response assessment [46,47].

Radiomics-based machine learning on multiparametric MRI shows strong capability for prostate cancer risk stratification and can outperform conventional image-reading approaches such as PI-RADS in several metrics, supporting more objective decision-making [48]. Random-forest classifiers trained on predominantly second-order radiomic features achieved AUC 0.87 for overall risk prediction and AUC 0.89 for identifying the high-risk group, highlighting the value of texture-level descriptors for clinically meaningful stratification [49].

Clinical integration of AI into prostate MRI workflows requires calibrated performance thresholds matched to the intended role (second-reader decision support, pre-screen triage, or more autonomous analysis) and explicit strategies to mitigate automation bias and erroneous outputs. For rule-out uses, high sensitivity and NPV are critical; for rule-in/confirmatory decisions, higher specificity and PPV are required. Prospective, real-world validation is essential before widespread deployment [50].

AI in histopathology and biomarker analysis

AI-based algorithms have transformed prostate biopsy assessment by automating cancer detection and Gleason grading with high accuracy. A recent systematic review report shows AI models outperform pathologists in detecting subtle cancer regions on whole-slide images [18]. Clinical-grade AI tools for prostate biopsy assessment show high accuracy on independent, real-world datasets and support more consistent grading across sites. In a three-center evaluation, a deployed system achieved a sensitivity of ~0.99 and a specificity of ~0.93, illustrating robust performance at scale [51]. AI assistance also reduces diagnostic workload-cutting reading time (~20%), immunohistochemistry (IHC) orders (~20%), and second-opinion requests (~40%) while maintaining pathologists’ accuracy [52]. A comprehensive systematic review concludes that AI for prostate histology delivers good-to-excellent diagnostic performance and can streamline slide screening; multiple included studies report accuracies exceeding 90% [53].

Advanced machine-learning models that integrate digital histology with molecular IHC (Ki-67) and clinicopathologic data improve prognostic stratification for biochemical recurrence beyond clinical models alone. In a cohort of radical prostatectomy patients, a deep-learning risk model combining H&E and Ki-67-stained images with clinical variables outperformed Cancer of the Prostate Risk Assessment post-Surgery (CAPRA-S) and Gleason-based approaches, reclassifying patients more accurately across risk groups. The pipeline leverages AI-driven quantification of IHC signals to augment biomarker evaluation and guide adjuvant-therapy decisions [54].

Emerging evidence indicates that AI-assisted analyses combining morphometric features from histology with genomic/biomarker data can improve the stratification of aggressive prostate cancer phenotypes. Studies report that such models help identify patients at higher risk of neuroendocrine differentiation or castration-resistant progression, offering signals that may inform more personalized therapy selection and follow-up strategies. Integrative AI frameworks that fuse digital pathology with molecular markers (e.g., Ki-67) and clinical variables further enhance prognostic assessment, complementing traditional risk models [54-56].

In an active-surveillance cohort, an AI biopsy-detection algorithm achieved a sensitivity of 0.96 and a specificity of 0.73, suggesting that large proportions of benign slides could be safely auto-screened, reducing pathologist workload while preserving accuracy [57]. Separately, a prospective study of a clinical-grade AI assistant in routine practice demonstrated efficiency improvements (shorter reading time, fewer ancillary tests) without loss of diagnostic accuracy, supporting digital workflow transition and quality assurance in high-volume laboratories [52].

AI-enabled biomarker discovery is accelerating the identification of molecular signatures linked to adverse outcomes by mining high-dimensional histology and omics data with robust feature selection and validation pipelines. Reviews of AI in pathology further show how automated image analysis and machine learning can enhance biomarker quantification and evaluation, supporting precision pathology and paving the way for patient-specific risk models and therapy optimization [2,53,54].

AI in treatment planning and optimization

AI-driven algorithms are transforming prostate cancer treatment planning, automating contouring and dose calculations for radiation therapy to improve efficiency, consistency, and clinical outcomes compared to manual workflows. Multicenter studies reveal AI-assisted planning can reduce planning time while maintaining high conformity indices [58,59].

Machine learning-based segmentation algorithms facilitate precise identification of organs at risk and tumor volumes, optimizing radiation delivery and sparing healthy tissue, as demonstrated in both external beam and brachytherapy settings. This automation supports reproducible and personalized treatment plans [58,59].

AI models help select and guide minimally invasive focal therapies, including high-intensity focused ultrasound (HIFU) and cryotherapy, by integrating MRI, clinical, and genomic data to improve candidate selection and ablation targeting, allowing tailored focal management for eligible patients. Automated imaging analytics enhance procedural accuracy [60, 61].

For surgical planning, deep learning algorithms analyze multiparametric MRI and histopathology to estimate tumor extent and surgical margins, supporting decisions for nerve-sparing or wider excisions, especially in robotic-assisted radical prostatectomies. This integration minimizes positive margin rates and preserves function [60].

Personalized systemic treatment regimens now rely on AI predictors of individual drug response and resistance, using multi-omics and clinical datasets to recommend optimal combinations and sequence adjustments for advanced prostate cancer patients. Adaptive machine learning models are aiding real-time therapy adjustment [62,63].

AI-assisted platforms monitor early post-treatment imaging and biomarkers for rapid detection of suboptimal responses or complications, triggering timely clinical intervention, an emerging protocol reducing recurrence risk and improving survival, as shown in recent feasibility trials. Continuous optimization marks a new era of dynamic prostate cancer management [62].

Limitations and future directions

AI shows promise for prostate cancer screening and diagnosis, but faces significant limitations. Multiple studies demonstrate that AI can achieve diagnostic accuracy comparable to expert radiologists and pathologists in detecting and grading prostate cancer on MRI and histopathology images [64,65].

However, systematic reviews reveal insufficient evidence for clinical deployment due to methodological flaws and evaluation biases [66]. Key limitations include variability in training datasets, algorithms, and evaluation criteria. Significant limitations emerge due to the lack of uniformity and agreement across various training datasets, the algorithms employed, and the metrics used for evaluation [65]. The effectiveness of AI in medical applications, particularly in the management of prostate cancer, is heavily influenced by the imaging systems employed. A critical observation is that the performance of AI models can vary significantly based on the specific technologies used for image acquisition. Furthermore, a major limitation in the current body of research is that validation studies predominantly rely on small, single-center cohorts. This narrow scope of data for validation means that the generalizability and robustness of AI models are not adequately tested across diverse multi-institutional datasets, which are essential for real-world clinical deployment [67].

Additional barriers include costly digital pathology workflows, lack of regulatory guidelines, and absence of prospective studies demonstrating clinical benefits [24]. The difficulty in verifying automatic prostate cancer system outputs due to a lack of clinically established test datasets further compounds these challenges [68].

Current AI models for prostate cancer often function as “black boxes,” where the reasoning behind diagnostic recommendations remains opaque to clinicians. This lack of transparency creates challenges for oncologists who must comprehend and justify treatment decisions to patients, potentially hindering clinical adoption of otherwise accurate AI systems [69].

AI algorithms in prostate cancer may perpetuate existing healthcare disparities if training datasets lack demographic diversity and representativeness. Models trained predominantly on specific population subgroups can demonstrate reduced performance when applied to underrepresented communities, potentially exacerbating health inequalities in cancer care [6].

Clinical deployment of AI systems faces significant workflow integration challenges, including expensive hardware requirements, software maintenance complexities, and the need for extensive staff training. These implementation barriers, combined with concerns about clinician over-reliance on automated systems, may diminish critical thinking skills essential for nuanced cancer treatment decisions [70].

## Conclusions

AI is reshaping the prostate cancer pathway, from risk-adapted screening and MRI/TRUS interpretation to digital pathology, PSMA-PET quantification, and automated radiotherapy planning. Across these domains, validated systems consistently raise diagnostic performance, streamline workflows, and support objective, reproducible decisions, while multimodal models add prognostic value that can guide focal, surgical, and systemic therapies. When deployed with clearly defined roles (triage, second reader, or autonomous modules), AI can reduce unnecessary biopsies, shorten reporting time, and enable more personalized care.

Realizing this promise requires rigorous, prospective, multi-center validation, harmonized datasets, and standardized reporting to address device dependence, spectrum bias, and generalizability gaps. Interoperable deployment within digital pathology and imaging ecosystems, governance for safety and equity, and post-market monitoring are essential. Future priorities include open benchmarks, calibration for intended use, integration of multi-omics and longitudinal data, and trials powered for patient-important outcomes and cost-effectiveness, ensuring trustworthy, human-in-the-loop AI that improves survival and quality of life.
